# Analysis of Inadequacies in Hospital Care through Medical Liability Litigation

**DOI:** 10.3390/ijerph18073425

**Published:** 2021-03-25

**Authors:** Raffaele La Russa, Rocco Valerio Viola, Stefano D’Errico, Mariarosaria Aromatario, Aniello Maiese, Paolo Anibaldi, Christian Napoli, Paola Frati, Vittorio Fineschi

**Affiliations:** 1 Department of Clinical and Experimental Medicine, University of Foggia, 71122 Foggia, Italy; raffaele.larussa@unifg.it; 2 IRCSS Neuromed Mediterranean Neurological Institute, Via Atinense 18, 86077 Pozzilli, Italy; paola.frati@uniroma1.it; 3 Department of Anatomical, Histological, Forensic and Orthopaedic Sciences, Sapienza University of Rome, Viale Regina Elena 336, 00161 Rome, Italy; roccovalerio.viola@uniroma1.it; 4 Department of Medicine, Surgery and Health, University of Trieste, Strada di Fiume 44, 34149 Trieste, Italy; sderrico@units.it; 5 Department of Medical and Surgical Sciences and Translational Medicine, Sapienza University of Rome, Via di Grottarossa 1035-1039, 00189 Rome, Italy; maromatario@ospedalesantandrea.it (M.A.); panibaldi@ospedalesantandrea.it (P.A.); christian.napoli@uniroma1.it (C.N.); 6 Department of Surgical Pathology, Medical, Molecular and Critical Area, University of Pisa, Ospedale Santa Chiara, Via Roma 55, 56126 Pisa, Italy; aniello.maiese@unipi.it

**Keywords:** health care litigation, claims, compensation, clinical inappropriateness, medical liability

## Abstract

Over the past two decades, health litigation has followed an exponentially incremental trend. As insurance companies tend to limit their interest because of the high risk of loss, health facilities increasingly need to internalize dispute management. This study was conducted through a retrospective analysis of existing files concerning the civil litigation of the Sant’Andrea Hospital in Rome. All claims from 1 June 2010 to 30 June 2019 were included. Paid claims were further classified according to the areas of health care inappropriateness found. Authors indexed 567 different claims along the study period, with an average number of 59 per year (range 38–77). The total litigation involved 47 different units; more than 40% concerned 5 high-incidence wards or services. Concerning the course of disputes, 91 cases were liquidated before a judicial procedure was instituted, while 177 cases landed in a civil court. Globally, 131 different claims hesitated in compensation, for a total of 16 million 625 thousand euros, 41% of which was related to the internal medicine area. Dealing with the inappropriateness analysis, clinical performance alone involved 76 cases, for a total of 10 million 320 thousand euros, while organization defects involved 20 disputes equivalent to 1 million 788 thousand euros. The aim of this study was to enhance the clinical risk management at our facility through a litigation analysis.

## 1. Introduction

In Italy, law number 24/2017 has modified the professional responsibility of the healthcare sector [1]. The right to safety of care is an integral part of the broader right to health [2]. Clinical risk management strategies are one of the tools that all healthcare professionals should adopt. The Joint Commission defines risk management in health care such as “the clinical and administrative activities undertaken to identify, evaluate, and reduce the risk of injury to patients, staff, and visitors and the risk of loss to the organization itself” [3]. The dispute for alleged malpractice is one of the specific issues of clinical risk management [4,5], because it is a potential source of economic damage for the healthcare facility [6]. The COVID-19 pandemic is a detrimental factor in this scenario [7,8,9,10].

The methods, tools and actions of risk management are part of the broader clinical governance [11]. Through this system, healthcare facilities are responsible for the continuous improvement of healthcare standards to facilitate the achievement of clinical excellence [12].

At the Sant’Andrea Hospital in Rome, there is a strong integration between monitoring, clinical audits, adverse event reporting, and litigation management activities [13]. This is made possible due to the implementation of the methodological and cultural principles of the correct application of clinical risk management criteria [14]. The multidisciplinary commission that embodies the strategic functions of the company in this area is the Hospital Claims Assessment Committee.

The objective of this study is to illustrate an operational model to integrate the reporting and analysis functions of medico-legal complaints and lawsuits directly into the risk assessment activities of clinical risk management, as suggested by the existing literature [15,16,17].

## 2. Materials and Methods

The study was conducted through the analysis of data collected along the activity carried out by the Claims Assessment Committee of Sant’Andrea Hospital in Rome; we did not need approval of an Ethics Committee. All claims from 1 June 2010 to 30 June 2019 were included. The study recognized 567 different claims reported from January 2010 to June 2019.

The various existing reports and datasets concerning the civil litigation of the hospital were brought together and standardized, using homogeneous descriptors. All the cases identified were integrated through direct consultation of the paper files. All the collected data we reorganized in a single database. First, we defined the categories of interest. We preferred categories suggested according to the International Classification for Patient Safety (ICPS) system, as for the incident type and the patient outcome (none; mild; moderate; severe; death). Next, we recorded the cases for which compensation payments occurred, and we further classified them according to the indications of the national scientific society of reference for hospital legal medicine about the areas of inappropriateness found. This medico-legal clinical practice discerns into three macro-areas: clinical performance; organization of health facilities; and law (including informed consent issues). Finally, we identified macro-areas according to the specificities of the hospital or health facility involved. The results were extracted from the database using synthetic calculation functions and coupling in pivot charts.

## 3. Results

### 3.1. Epidemiological Data

The Sant’Andrea hospital in Rome is a university hospital with 395 beds for a total of 22,000 annual hospitalizations with more than 50,000 people treated in the emergency department.

**Figure 1:**
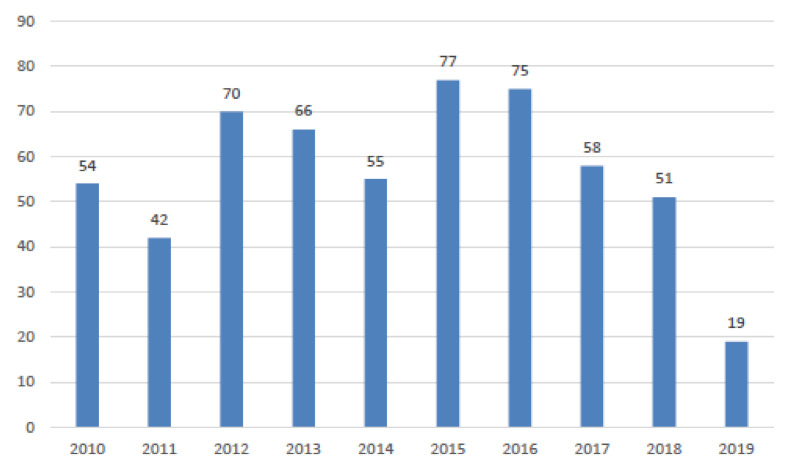
New claims per year (2019 represents data for the first six months).

The average number of new claims per year was 59, distributed in a range from 38 to 77. Claims remained almost constant in the five-year period of 2012–2016 to subsequently assume a decreasing trend, as shown in Figure 1.

**Figure 2:**
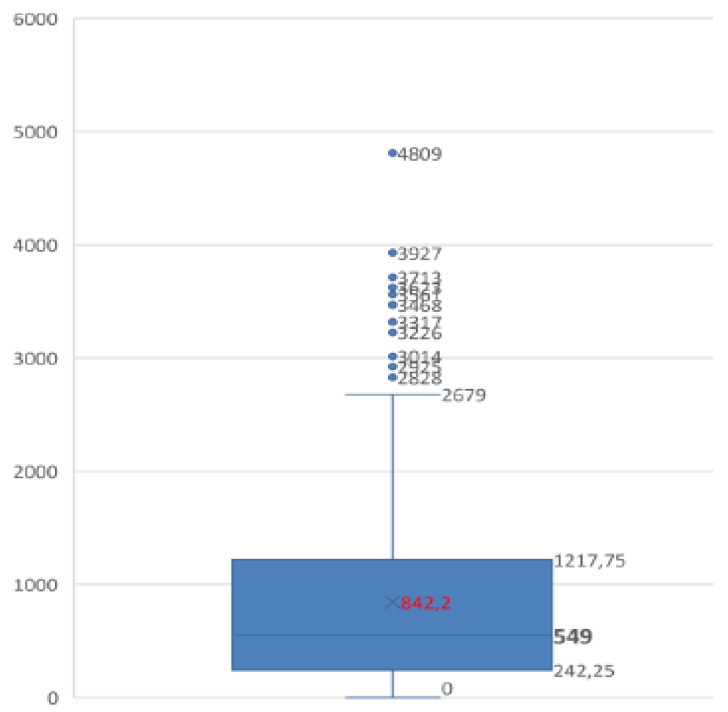
Box plot of claims latency.

**Figure 3:**
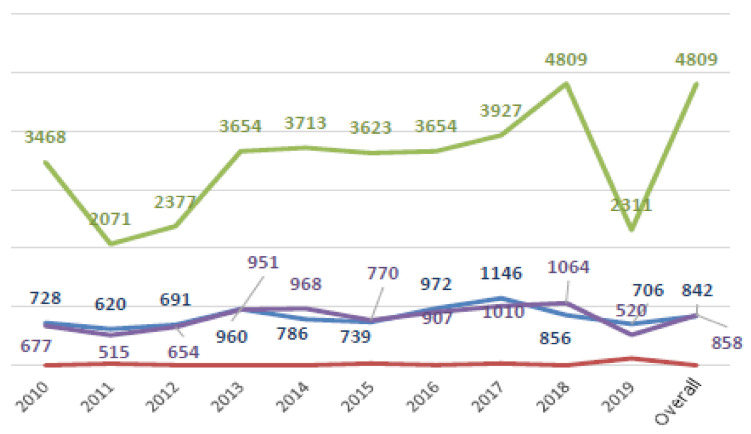
Latency trends by year (average in blue; minimum in red; maximum in green; standard deviation in violet).

The evaluation of the latency time between the date of the event and the date of the complaint showed a mean value of 28 months (range 1–160), highlighting relative stability during the study period. Regarding this aspect, it was also noted that 50% of total claims were reported within18 months from the date of the adverse event, while 98% of complaints occurred within the 89 months (7 years and a half); see Figure 2 and Figure 3 for trends by year of claims latency.

### 3.2. Demographic Data and Units Involved

The patients involved were characterized by sex and age corresponding to the date of the harmful event. The mean age was 55 years (range: 1–89), while the distribution for sex was perfectly balanced (283 female and 284 male subjects). In 80% of the claims, it was possible to identify the single clinical unit of reference. The total litigation was, thus, distributed over 47 different units, but more than 40% concerns five high-incidence wards or services: orthopedics and traumatology; emergency department; general surgery; neurosurgery; and radiology (see Table 1).

**Figure 4:**
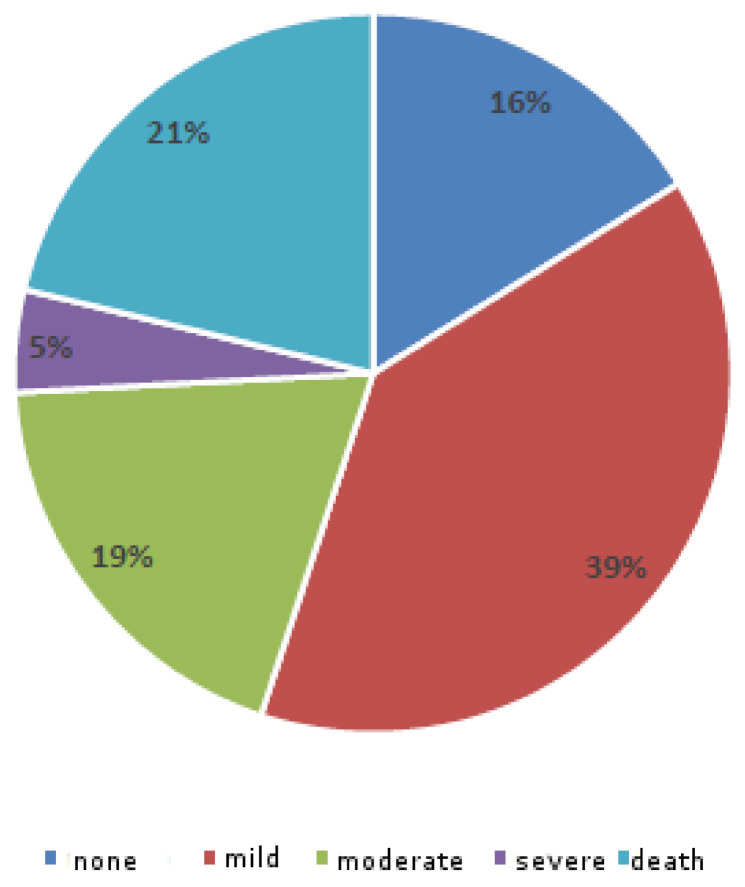
Patient outcome for claims (according to the International Classification for Patient Safety degree of harm).

Following the classification by patient outcome according to the ICPS system, it was possible to distribute all cases among five degrees of harm: none; mild; moderate; severe; and death (see Figure 4).

### 3.3. The Claims

The course of claims was reconstructed concerning the outcome of the disputes. Out of a total of 567 claims for compensation, 91 were compensated before a judicial procedure was instituted. Of the remainder, 177 cases have landed in a civil court, most of which are still pending. Compared to the claims included in the study period that progressed to the judicial stage, 29 were extinguished following a compensation payment; for 17 of them, the payment followed an explicit sentence by the judge.

From the data collected, a judicial conversion rate for unacknowledged claims of 37.2% is deduced. The analysis of the identifiable areas of clinical inappropriateness was conducted solely on the claims for which compensation for any reason was paid by the hospital itself or by the insurance company. The study included 131 different claims for a total of 16,625,000 euros in compensation with an average unit cost of approximately 127 thousand euros. The insurance company has borne most of the economic burden, both in terms of number (95 claims against 35, while one claim was shared) and cumulative amounts (13,250,000 euros against 3,374,000 euros).

Due to the specific nature of the insurance contract (deductible thresholds and aggregate ceilings), 86% of the claims paid by the company were granted before the initiation of a civil judgment, against 28% sustained directly by the hospital. Considering the cumulative amounts, the compensation paid directly by the hospital before the judgment is initiated counts for only 4% of the total, compared to 59% of the insurance company’s disbursements. These differences are inevitably reflected in the time elapsed between the initiation of the litigation and payment, which corresponds to 13 months for the insurance company against the 4 years and 3 months of the hospital.

The number of claims resulting in compensation reflects a similar imbalance between surgery and internal medicine as observed in general claims (59% against 18% of the total). This difference, however, is less pronounced in the calculation of the cumulative amounts, almost equally distributed between 48% of the surgical area and 41% of internal medicine. Conversely, the scope of general civil liability concerns less than 1% of the compensation amounts, although it concerns 11% of the total claims.

Of the 131 claims compensated, 22 were excluded from the inappropriateness analysis, equal to approximately 600 thousand euros cumulative. The claims included were, thus, distributed in the various macro-areas. Clinical performance alone involved 76 claims, for a total of 10 million 320 thousand euros in compensation, while defects attributable to the organization of the healthcare facility or the single operating unit involved 20 disputes equivalent to 1 million 788 thousand euros. For only six cases, though corresponding to 2 million 943 thousand euros, both clinical and organizational inappropriateness were identified.

Finally, problems relating to informed consent were found in the remaining seven claims, for a total of approximately 1 million 250 thousand euros, alone or combined with clinical performance or organization issues. Within clinical performance and organizational problems, subcategories of inappropriateness have been indexed, as shown in Table 2 and Table 3.

In the cases examined for which a technical error occurred as an element of health liability, the assistance service in question systematically provided for the execution of a surgical act. The case of the non-application of scientific evidence, on the other hand, was67% of the cumulative amounts only in the internal medicine area.

The problem of healthcare-associated infections (HAI) and shortcomings in prevention measures absorb 85% of the amounts attributed to organizational failures. Furthermore, it alone involves 10% of the claims in question and 25% of the amount of compensation paid. A detailed mapping of the cases between the different operating units was carried out, identifying a prevalence of the surgical area for 75% (see Table 4 for more details).

Invasive procedures represented among the compensated claims were ordered, comparing both the number and, above all, the specific economic impact. Of the 47 different procedures, we report the 10 most important both in terms of claims number and compensation amounts through two comparative tables (see Table 5 and Table 6).

## 4. Discussion

This study presents 567 general claims from 1 January 2010 to 30 June 2019, relevant for damages received by the hospital or the insurance company as civil liability. The ratio between claims for damages and the number of hospitalizations in the reviewed period is 26.8. In 2015, 77 claims were registered, with a gradual decrease in subsequent years, up to 2019, with only 40 civil suits. In 2019, a reduction in claims of 20% compared to the previous year was evident. Based on the ICPS indicators, the fatal events are frequent (one-fifth of the total), as opposed to a slight prevalence (55% of the total) of events with minimal or no permanent impairment. About 11% of all claims are attributable to general civil liability. This study examines the history of the claims, the acceptance and out-of-court settlement or the rejection, and the possible activation of a civil judgment. This examination is overall on annual basis (no reported data). In total, 75% of claims were settled before the civil sentence. However, considering the cumulative compensation amounts, extrajudicial payments represent less than half (48%). The management by the insurance company was different from that of the hospital. Indeed, the company has compensated out of court in 60% of the claims, unlike the hospital. The analysis of the malpractice claims highlighted a greater involvement of the clinical areas. The prevalence of the surgical branches is minimal compared to the clinical wards, in particular with internal medicine (41% of the total amount).

This value is higher compared to most of the series published in the literature [18], and the difference is even more evident considering the years with the highest incidence in the period 2012–2015. The management policy of the operating insurance company could be one of the factors influencing these data.

The deflationary trend of general claims observed in the period after 2015 for the Sant’Andrea Hospital contrasts with the data reported in the specialized literature during the same period [19]. The annual report published by the international insurance brokerage company Marsh in 2018 documented an incremental trend in health claims in Italy [20]. The contrast between the data of this study and the Italian data is difficult to interpret.

The reduction in complaints may be due to both improved clinical standards and/or increased caution of health professionals. This type of evaluation is beyond the scope of this study. The analysis of the temporal distance between the verification of the damage and the malpractice claims is difficult to interpret due to the high statistical dispersion. The trend over time of the variance measures, however, shows some stability over the study period. Similarly, the incidence of claims in the most involved operating units is constant (no reported data). The data are homogeneous compared to the literature data analyzing the frequency of complaints in the risk wards [21]. There is a prevalence of surgical specialties (in particular orthopedics, general surgery, and neurosurgery) and first aid; instead, the diagnostic services are scarcely involved. The low incidence of malpractice claims in the gynecological ward, apparently contrary to the general evidence [22], depends on the absence of a birth care unit at Sant’Andrea Hospital.

However, the peculiarity of this study is the in-depth analysis of the progress of the malpractice risk analysis, such as the examination of management strategies and the analysis of the amount paid [23].

The management of the insurance company (before the litigation) influenced the settlement rate during the period 2012–2016. The general judicial conversion rate of unpaid claims, on the other hand, is affected by the short time interval since the opening of the claims in the last two years of the examined period (2018 and 2019). Therefore, the value of 37.2% represents an underestimated value, considering that the annual range will vary from 47.5% in 2015 to 10% in 2019. Studying the compensation, considering the number of claims and the amounts paid, made it possible to better understand the characteristics of the management by the hospital and the insurance company.

Neurosurgical complaints are fewer than orthopedic ones, but they bring higher compensation, since the damages are more serious. Similarly, radiology and civil liability complaints lead to low compensation, despite the large number. According to the published data about the distribution of health disputes, medical and surgical areas are not generalizable because every specialty, every health service, and every hospital hasits peculiarities [24]. The number of disputes, especially the catastrophic ones, is hardly predictable.

This analysis was integrated through the inadequacy for macro-areas and sub-categories of eligible paid claims, corresponding to 96.4% of the total amount. This management tool reflects some peculiarities of the understudied hospital. The prevalence of clinical complaints is due to the increased surgical tendency of the hospital, both for the distribution of specialists, beds, and hospitalizations. Furthermore, the university nature of the hospital plays an important role; there is an increased concentration of complex cases with the expectation of excellent results [25].

The management of healthcare-associated infections is a fundamental problem, which increases litigation. This study confirms the relevance of the healthcare-associated infections in clinical risk management, as reported by other authors [26,27,28]. Breach of informed consent disputes were few in this case series, unlike reported elsewhere [29,30].

We acknowledge three main limitations of our study. First, it accounts for a relatively short time interval. The reform introduced with the 2017 law could produce profound changes in the system of medical professional liability that will only occur in the future. Second, many of the disputes included in the study are still pending in court; thus, it was not possible to assess their compensation. Third, our study is based on the claims made by allegedly injured patients without considering the health services actually provided and the clinical outcomes obtained. This last limitation could be a source of considerable bias with respect to the qualitative evaluation of the services provided; for this reason, we believe that future investigations will be necessary to correlate global clinical outcomes with litigation.

## 5. Conclusions

This study constitutes a detailed analysis of the malpractice claims of the hospital Sant’Andrea in Rome over 10years. This study extends the analysis to the longitudinal trend of claims, from the request submission to any compensation. Another aspect is the enlightenment of a management tool for the inappropriateness mapping, never applied in scientific publications to the best of our knowledge. The medical-legal study of claims proves to be a useful tool to integrate clinical risk management activities [31]. The data collection increased incident reporting resources and potential weakness identification to be analyzed to make the clinical care processes safe [32].

Finally, this study underlines the importance of the clinical risk manager specialist for the management of malpractice claims. The analysis of malpractice claims data allows the detection of the patterns and the consequences of iatrogenic errors; the risk manager can improve safety in a more targeted manner [33].

## Figures and Tables

**Table 1:** High-incidence wards or services (general claims).

Ranking	High-Incidence Departments	% Of Suitable Claims
1	Orthopedics and traumatology	16.6
2	Emergency department	13.3
3	General surgery	11.4
4	Neurosurgery	7.7
5	Radiology	3.7

**Table 2:** Clinical performance inappropriateness.

Clinical Performance Sub-Category	Claims Numbers	Total Amounts (Euros)
Disapplication scientific evidences	11	6,714,000
Technical error	65	5,614,000
Missed or delayed diagnosis	11	1,904,000
Overall	87	14,233,000

**Table 3:** Service organization inappropriateness.

Service Organization Inappropriateness	Claims Numbers	Total Amounts (Euros)
Specific procedure violation	11	300,000
Poor Health-care associated infection (HAI) prevention	12	4,157,000
Fall prevention violation	4	43,000
Suicide prevention violation	1	370,000
Overall	28	4,871,000

**Table 4:** HAI: distribution of claims number and compensation amount among operating units.

Operating Units	Claims Number (%)	Compensation Amount (%)
Orthopedics	9	0
Internal medicine	8	5
Urology	17	9
General surgery	17	10
Cardiac surgery	8	10
Neurosurgery	17	15
Cardiology	8	16
Vascular surgery	8	17
Neurology	8	18

**Table 5:** Claims number per kind of invasive procedure.

Procedure	Claims Number	Ranking per Cumulative Amount
oro-tracheal intubation	5	28th
lumbar spine surgery (elective)	4	4th
radioulnar fracture treatment	4	20th
unilateral kidney surgery	3	6th
thyroidectomy (non-cancer)	3	12th
thyroidectomy (oncological)	3	14th
breast reconstruction	2	13th
ovarian surgery	2	18th
dental extraction	2	41st
all the others	1	-

**Table 6:** Compensation amount per kind of invasive procedure.

Procedure	Compensation Amount	Claims Number
tumor exeresis through craniotomy	787,000	1
aortic valve replacement	600,000	1
hemodialysis	530,000	1
lumbar spine surgery (elective)	446,200	4
trans-sphenoid tumor exeresis	377,000	1
unilateral kidney surgery	362,900	3
colorectal resection (elective)	268,000	1
shoulder arthroplasty	265,300	1
endovascular stenting	228,200	1
mandibular reconstruction	182,000	1

## Data Availability

Data supporting reported results are available on request.

## References

[1] Albolino S., Bellandi T., Cappelletti S., Di Paolo M., Fineschi V., Frati P., Offidani C., Tanzini M., Tartaglia R., Turillazzi E. (2019). New Rules on Patient’s Safety and Professional Liability for the Italian Health Service. Curr. Pharm. Biotechnol..

[2] Bellandi T., Tartaglia R., Sheikh A., Donaldson L. (2017). Italy recognises patient safety as a fundamental right. BMJ.

[3] (2007). The Joint Commission releases improving America’s hospitals: The Joint Commission’s annual report on quality and safety 2007. Jnt. Comm. Perspect..

[4] Hsieh S.Y. (2010). The use of patient complaints to drive quality improvement: An exploratory study in Taiwan. Health Serv. Manag. Res..

[5] Mello M.M., Studdert D.M. (2016). Building a National Surveillance System for Malpractice Claims. Health Serv. Res..

[6] Greve P.A. (2002). Anticipating and controlling rising malpractice insurance costs. Healthc. FinancManag..

[7] Riley-Smith Q.T., Heppinstall A., Foster F. (2020). Is Covid-19 sowing the seeds for future litigation?. Med. Leg. J..

[8] Bilotta C., Zerbo S., Perrone G., Malta G., Argo A. (2020). The medico-legal implications in medical malpractice claims during Covid-19 pandemic: Increase or trend reversal?. Med. Leg. J..

[9] Oliva A., Caputo M., Grassi S., Vetrugno G., Marazza M., Ponzanelli G., Cauda R., Scambia G., Forti G., Bellantone R. (2020). Liability of Health Care Professionals and Institutions During COVID-19 Pandemic in Italy: Symposium Proceedings and Position Statement. J. Patient Saf..

[10] Pagano A.M., Maiese A., Izzo C., Maiese A., Ametrano M., De Matteis A., Attianese M.R., Busato G., Caruso R., Cestari M. (2020). COVID-19 Risk Management and Screening in the Penitentiary Facilities of the Salerno Province in Southern Italy. Int. J. Environ. Res. Public Health.

[11] Piccioni A., Cicchinelli S., Saviano L., Gilardi E., Zanza C., Brigida M., Tullo G., Volonnino G., Covino M., Franceschi F. (2020). Risk management in first aid for acute drug intoxication. Int. J. Environ. Res. Public Health.

[12] La Russa R., Fineschi V., Di Sanzo M., Gatto V., Santurro A., Martini G., Scopetti M., Frati P. (2017). Personalized Medicine and Adverse Drug Reactions: The Experience of an Italian Teaching Hospital. Curr. Pharm. Biotechnol..

[13] Ivers N., Jamtvedt G., Flottorp S., Young J.M., Odgaard-Jensen J., French S.D., O’Brien M.A., Johansen M., Grimshaw J., Oxman A.D. (2012). Audit and feedback: Effects on professional practice and healthcare outcomes. Cochrane Database Syst. Rev..

[14] Mirzoev T., Kane S. (2018). Key strategies to improve systems for managing patient complaints within health facilities—What can we learn from the existing literature?. Glob. Health Action.

[15] Jonsson P.M., òvretveit J. (2008). Patient claims and complaints data for improving patient safety. Int. J. Health Care Qual. Assur..

[16] Bishop T.F., Ryan A.M., Casalino L.P. (2011). Paid malpractice claims for adverse events in inpatient and outpatient settings. JAMA.

[17] Javetz R., Stern Z. (1996). Patients’ complaints as a management tool for continuous quality improvement. J. Manag. Med..

[18] Bonetti M., Cirillo P., MusileTanzi P., Trinchero E. (2016). An Analysis of the Number of Medical Malpractice Claims and Their Amounts. PLoS ONE.

[19] National Association of Insurance Companies (ANIA) Executive Summary 2015–2016. www.ania.it/InsuranceInsurance/2015–2016/ANIA-Italian-Insurance-2015-16.pdf.

[20] Marsh Risk Consulting (2018). Report Medmal. Annual Report About Medical Malpractice Trends in Private and Public Health Facilities in Italy.

[21] Studdert D.M., Mello M.M., Gawande A.A., Gandhi T.K., Kachalia A., Yoon C., Puopolo A.L., Brennan T.A. (2006). Claims, errors, and compensation payments in medical malpractice litigation. N. Engl. J. Med..

[22] Adinma J. (2016). Litigations and the Obstetrician in Clinical Practice. Ann. Med. Health Sci. Res..

[23] Zerbo S., Malta G., Argo A. (2020). Guidelines and Current Assessment of Health Care Responsibility in Italy. Risk Manag. Healthc. Policy.

[24] Levin C.M., Hopkins J. (2014). Creating a Patient Complaint Capture and Resolution Process to Incorporate Best Practices for Patient-Centered Representation. Jnt. Comm. J. Qual. Patient Saf..

[25] Leape L.L. (1994). Error in medicine. JAMA.

[26] Giraldi G., Montesano M., Napoli C., Frati P., La Russa R., Santurro A., Scopetti M., Orsi G.B. (2019). Healthcare-Associated Infections Due to Multidrug-Resistant Organisms: A Surveillance Study on Extra Hospital Stay and Direct Costs. Curr. Pharm. Biotechnol..

[27] Siracusa M., Scuri S., Grappasonni I., Petrelli F. (2019). Healthcare acquired infections: Malpractice and litigation issues. Ann. Ig..

[28] Albano G.D., Bertozzi G., Maglietta F., Montana A., Di Mizio G., Esposito M., Mazzeo P., D’Errico S., Salerno M. (2019). Medical Records Quality as Prevention Tool for Healthcare-Associated Infections (HAIs) Related Litigation: A Case Series. Curr. Pharm. Biotechnol..

[29] D’Errico S., Pennelli S., Colasurdo A.P., Frati P., Sicuro L., Fineschi V. (2015). The right to be informed and fear of disclosure: Sustainability of a full error disclosure policy at an Italian cancer centre/clinic. BMC Health Serv. Res..

[30] Cartocci G., Santurro A., La Russa R., Guglielmi G., Frati P., Fineschi V. (2017). The choice of gadolinium-based contrast agents: A radiologist’s responsibility between pharmaceutical equivalence and bioethical issues. Symmetry.

[31] Bolcato M., Fassina G., Rodriguez D., Russo M., Aprile A. (2019). The contribution of legal medicine in clinical risk management. BMC Health Serv. Res..

[32] Yarmohammadian M.H., Abadi T.N., Tofighi S., Esfahani S.S. (2014). Performance improvement through proactive risk assessment: Using failure modes and effects analysis. J. Educ. Health Promot..

[33] Gatto V., Scopetti M., La Russa R., Santurro A., Cipolloni L., Viola R.V., Di Sanzo M., Frati P., Fineschi V. (2019). Advanced Loss Eventuality Assessment and Technical Estimates: An Integrated Approach for Management of Healthcare-Associated Infections. Curr. Pharm. Biotechnol..

